# A Three-Tiered Study of Differences in Murine Intrahost Immune Response to Multiple Pneumococcal Strains

**DOI:** 10.1371/journal.pone.0134012

**Published:** 2015-08-05

**Authors:** Ericka Mochan-Keef, David Swigon, G. Bard Ermentrout, Gilles Clermont

**Affiliations:** 1 Joint Carnegie Mellon University-University of Pittsburgh PhD Program in Computational Biology, Pittsburgh, PA, United States of America; 2 Department of Mathematics, University of Pittsburgh, Pittsburgh, PA, United States of America; 3 Department of Critical Care Medicine, University of Pittsburgh, Pittsburgh, PA, United States of America; 4 McGowan Institute for Regenerative Medicine, Center for Inflammation and Regenerative Modeling, University of Pittsburgh, Pittsburgh, PA, United States of America; Universidad Nacional de La Plata., ARGENTINA

## Abstract

We apply a previously developed 4-variable ordinary differential equation model of in-host immune response to pneumococcal pneumonia to study the variability of the immune response of MF1 mice and to explore bacteria-driven differences in disease progression and outcome. In particular, we study the immune response to D39 strain of bacteria missing portions of the pneumolysin protein controlling either the hemolytic activity or complement-activating activity, the response to D39 bacteria deficient in either neuraminidase A or B, and the differences in the response to D39 (serotype 2), 0100993 (serotype 3), and TIGR4 (serotype 4) bacteria. The model accurately reproduces infection kinetics in all cases and provides information about which mechanisms in the immune response have the greatest effect in each case. Results suggest that differences in the ability of bacteria to defeat immune response are primarily due to the ability of the bacteria to elude nonspecific clearance in the lung tissue as well as the ability to create damage to the lung epithelium.

## Introduction

*Streptococcus pneumoniae*, known as pneumococcus, continues to be a leading cause of morbidity and mortality worldwide, particularly in children and the elderly. Pneumococci, which are virulent encapsulated bacteria, are the most common cause of bacterial pneumonia [1]. The capsule of these bacteria, composed almost entirely of polysaccharides, shields the bacteria from several host defense mechanisms and contributes significantly to the virulence of pneumococci [2–4]. In fact, encapsulated strains are about 100,000 times more virulent than strains without a capsule [5,6].

The virulence of encapsulated bacteria depends on capsule thickness and chemical composition. A thicker capsule is advantageous in that it allows the bacteria to evade phagocytosis by immune cells such as neutrophils or macrophages [7]. However, thicker capsules also impede the ability of the bacteria to migrate from lung tissue to the bloodstream, decreasing their ability to cause bacteremia [8]. Bacteria in blood tend to proliferate faster than in tissue [9,10], and the number of activated phagocytes in the blood is proportionally lower than the number which migrate into infected tissue. Thus, even though a thinner capsule leaves the bacteria more vulnerable to phagocytosis, a thin capsule may also be advantageous for survival in some host species. Because of this dichotomy, pneumococcal serotypes have evolved with a range of capsule thicknesses.

In addition to capsule thickness, the chemical composition of the capsule is significant to bacterial fitness. Serotypes of pneumococcus are distinguished by the presence of specific glycoprotein motifs present on the surface of the capsule that influence the activation of the complement [11,12], degradation of complement components [13], and resistance to phagocytosis [14]. To date, over 90 serotypes of pneumococcus have been identified [15]. Each serotype can induce a different reaction from the immune system, depending on the makeup of the capsule and the activity of virulence factors associated with the bacterial surface.

Aside from the capsule, there are additional virulence factors, present both on the surface and within the bacterium, which play an important role in the ability of the bacteria to evade the immune system. Examples of such virulence factors are pneumolysin and neuraminidase. Pneumolysin is a pore-forming toxin expressed by virtually all serotypes of pneumococcus [16]. In early stages of the infection, when bacteria exist in low levels in the body, pneumolysin is cytotoxic, causing apoptotic activity in the epithelial cells [17] and activation of the complement system [18]. As the infection progresses and pneumolysin concentration reaches higher levels, it is lytic to any cell with cholesterol in the membrane [19]. This lytic action causes damage to the tissue, and surrounding epithelial cells will increase the cytokine signaling to activate immune cells. In the absence of pneumolysin, the influx of phagocytic cells is delayed and decreased [20]. Neuraminidase is a surface protein that promotes the attachment of the bacteria to the epithelium. Neuraminidase cleaves the terminal sialic acid from glycolipids and glycoproteins, which damages the epithelium, exposes more potential binding sites for the bacteria, and promotes colonization [21,22]. Pneumococci have two neuraminidases on their surfaces: neuraminidase A (NanA) and neuraminidase B (NanB) [23]. Though bacteria deficient in either NanA or NanB are unable to cause sepsis in mice, NanA-deficient (NanA^−^) bacteria have been shown to cause less damage than NanB^−^ bacteria [23].

The present study explores differences in host response to pneumococcus infection of the lung for a selected number of pneumococcal serotypes and virulence factors using an ordinary differential equation (ODE) model described in our earlier work [24]. We have previously shown this model to accurately predict bacteria levels in the lungs and blood of four strains of mice (CBA/Ca, MF1, BALB/c, C57BL/6) inoculated with an identical strain of bacteria (D39). We now present a complementing study, showing our model can accurately predict bacterial behavior for a single strain of mouse (MF1) infected with one of several different types of pneumococci. We first explore the response to D39 (serotype 2) bacteria missing portions of the pneumolysin protein controlling either the hemolytic activity (H2-/C+) or complement-activating activity (H+/C-) [25]. Next, we model the response to D39 bacteria deficient in either NanA or NanB [23]. Lastly, we explore the response to three different serotypes of pneumococcus: D39 (serotype 2), 0100993 (serotype 3), and TIGR4 (serotype 4) bacteria [26]. Notably, a change in few key parameters expressing the activity of immune response components and virulence factors in our 4-variable ODE model captures the differences in the progression of infection exhibited by each phenotype of bacteria.

## Results

### Overview of modeling strategy

The ODE model used in this study was first presented in Mochan *et al*., 2014 [24]. The model follows the time-dependent trajectories of bacterial populations in both the lung (*P*_*L*_) and the blood (*P*_*B*_) compartments, damage to the epithelial cells lining the lung (*D*), and total activated phagocytic cells levels (*N*). These populations are modeled with 4 differential equations that depend on 17 parameters. In each of our three studies (pneumolysin activity study, neuraminidase study, and serotype study), we define a subset of the model parameters as “bacterial-strain-dependent”. These parameters are chosen based on existing knowledge of the mechanisms of immune response regulation so as to account for expected phenotypic differences seen in the progression of disease caused by each strain of bacteria modeled in the study. All other parameters are defined as “bacterial-strain-independent”; these parameters, which include parameters governing phagocyte lifespan, bacteria growth rates, threshold parameters, and parameters inherent to host tissue, are assumed not to differ across strains of bacteria. Table 1 summarizes the bacteria-strain-dependent parameters for all three sub-studies.

**Table 1:** Summary of bacteria-strain-dependent parameters in each of the three studies presented.

Study	Parameters varied between strains	Reference
Pneumolysin activity	*h*, *q*, *ν*, *ξ*_*nl*_, *ξ*_*nb*_	[25]
Neuraminidase	*q*, *ν*, *a*, *ξ*_*nl*_, *ξ*_*nb*_	[23]
Serotype	*h*, *q*, *a*, *ν*, *ξ*_*nl*_, *ξ*_*nb*_	[26]

Though each study focuses on different types of bacteria, our method for analyzing the data is uniform across all three studies. First, we estimate the uncertainty in parameter values by using a Bayesian inference approach coupled with Metropolis-Hastings Monte Carlo sampling, and compute a posterior distribution in parameter space. This posterior distribution forms a model ensemble, a collection of generated parameter sets compatible with both the data and the biological heuristic constraints. Then, we compute the ensemble of trajectories fitted to the data for each type of bacteria, and full marginal posterior distributions for all bacteria-strain-dependent parameters, illustrating the primary causes for differences in immune responses seen for each bacterial phenotype. Finally, we use singular value decomposition of each ensemble to describe the eigenvectors. Important components to the eigenvector associated with the smallest eigenvalue identify parameters that are key drivers of the biological response.

### Pneumolysin activity study

In the study of Jounblat *et al*., [25], female MF1 mice were infected with D39 bacteria to study the impact of pneumolysin on the survival of bacteria in the body. The study involved wild-type D39 and two mutant strains: H+/C-, in which pneumolysin lacks its complement-activating activity, and H2-/C+, in which pneumolysin has substantially reduced hemolytic (pore-forming) activity. The C location on the pneumolysin protein activates the classical complement pathway [27]. Decreased activation of complement leads to decreased phagocytic activity in both compartments and decreased activation of nonspecific immunity. In our model, these effects are controlled by *h*, the rate at which phagocytes are activated by lung bacteria; *ν*, the rate of nonspecific clearance of bacteria from the lungs by mucociliary clearance and resident macrophages; *ξ*_*nl*_, the rate at which phagocytes clear bacteria from the lungs; and *ξ*_*nb*_, the rate at which phagocytes clear bacteria from the blood. The H segment of the pneumolysin controls the hemolytic activity of the bacteria, and hence the rate at which damage to the epithelium increases due to the presence of bacteria in the lungs, which is in our model represented by parameter *q*, We therefore fit the model to the pneumolysin study data by allowing only *h*, *ν*, *ξ*_*nl*_, *ξ*_*nb*_, and *q* to vary across the bacterial strains.

**Fig 1:**
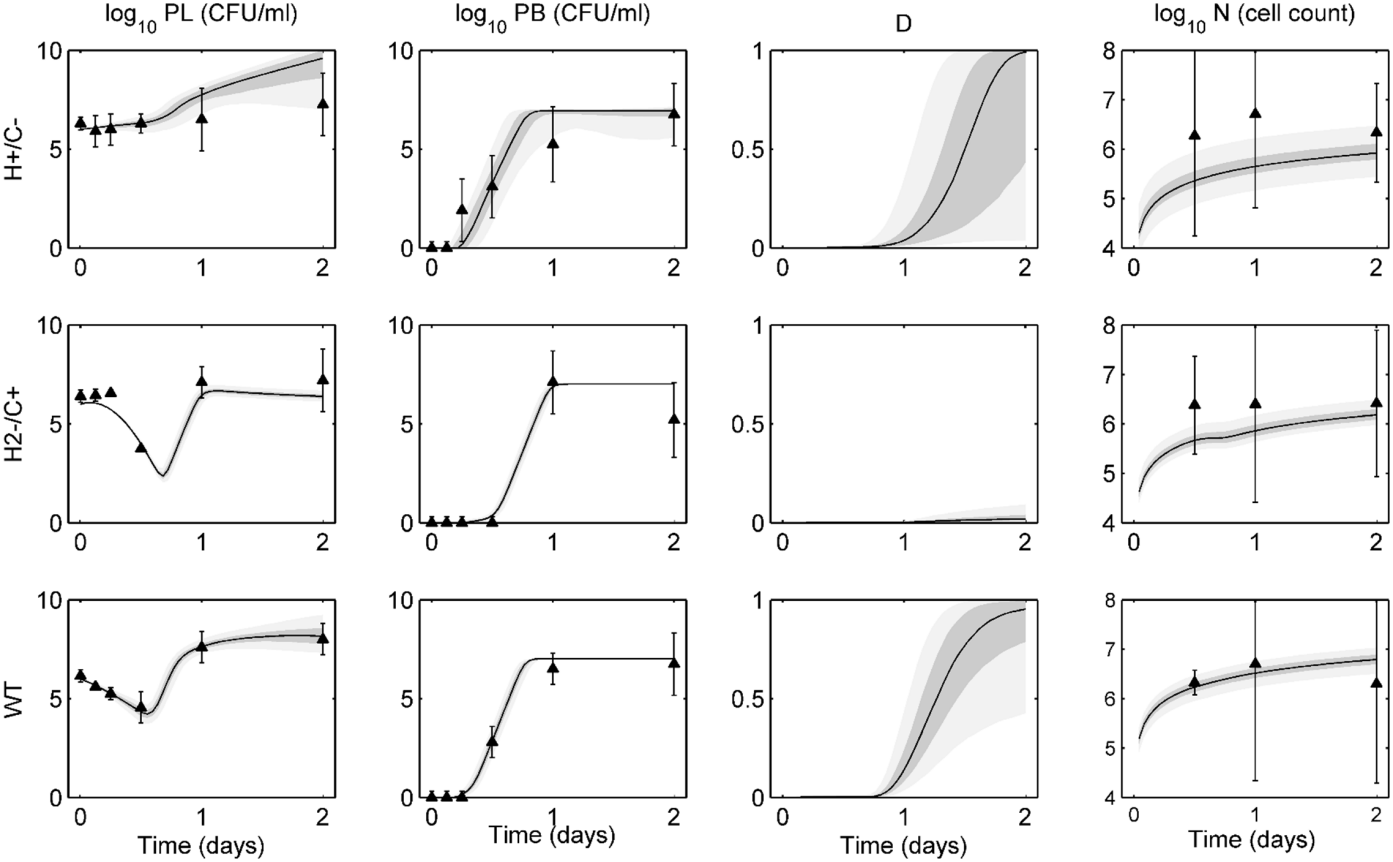
Ensemble trajectories of pneumolysin activity study. Ensemble fits of each strain for lung pathogen (*P*_*L*_), blood pathogen (*P*_*B*_), epithelial damage (*D*), and activated phagocytic cells (*N*). The black line represents the median trajectory, the inner dark gray area represents the 25^th^ to 75^th^ quantiles of trajectories, and the outer light gray envelope represents 90% of the trajectories (5^th^ to 95^th^ quantiles). Data points with standard deviations are represented by the black triangles with error bars. Data were taken at 0, 3, 6, 12, 24, and 48 hours post-infection with ten mice in each group. Trajectories are simulated over two days, with infection occurring on day 0. The top row shows ensembles for H+/C- bacteria, the middle row shows ensembles for H2-/C+ bacteria, and the bottom row shows ensembles for the wild-type (WT) bacteria.

Fig 1 displays the ensemble fits to data for wild-type, H+/C-, and H2-/C+ D39 bacteria. On each ensemble trajectory plot, we represent the median trajectory as a solid black line, with the 25–75% quantiles in dark gray and 5–95% percentiles in light gray. Mean experimental data are represented by the black triangles, with standard deviations presented by the error bars.

In both lung and blood, each strain exhibits distinct behavior in the first 12 hours post-infection. The H+/C- bacteria stay at a near constant high level for the first 12 hours and reach the bloodstream in only 2 hours. The H2-/C+ bacterial population remains essentially level in the lungs until a sharp decrease at 12 hours, while showing negligible levels in the blood during that time. Bacterial populations in both compartments then begin to rise quickly, as transport between compartments increases and the bacteria can more easily avoid the immune system. The wild-type bacteria exhibit a gradual, steady decline in lung population levels. Wild-type bacteria reach the blood in about 6–8 hours post-infection. As Fig 1 shows, the model captures all these behaviors within the ensemble.

**Fig 2:**
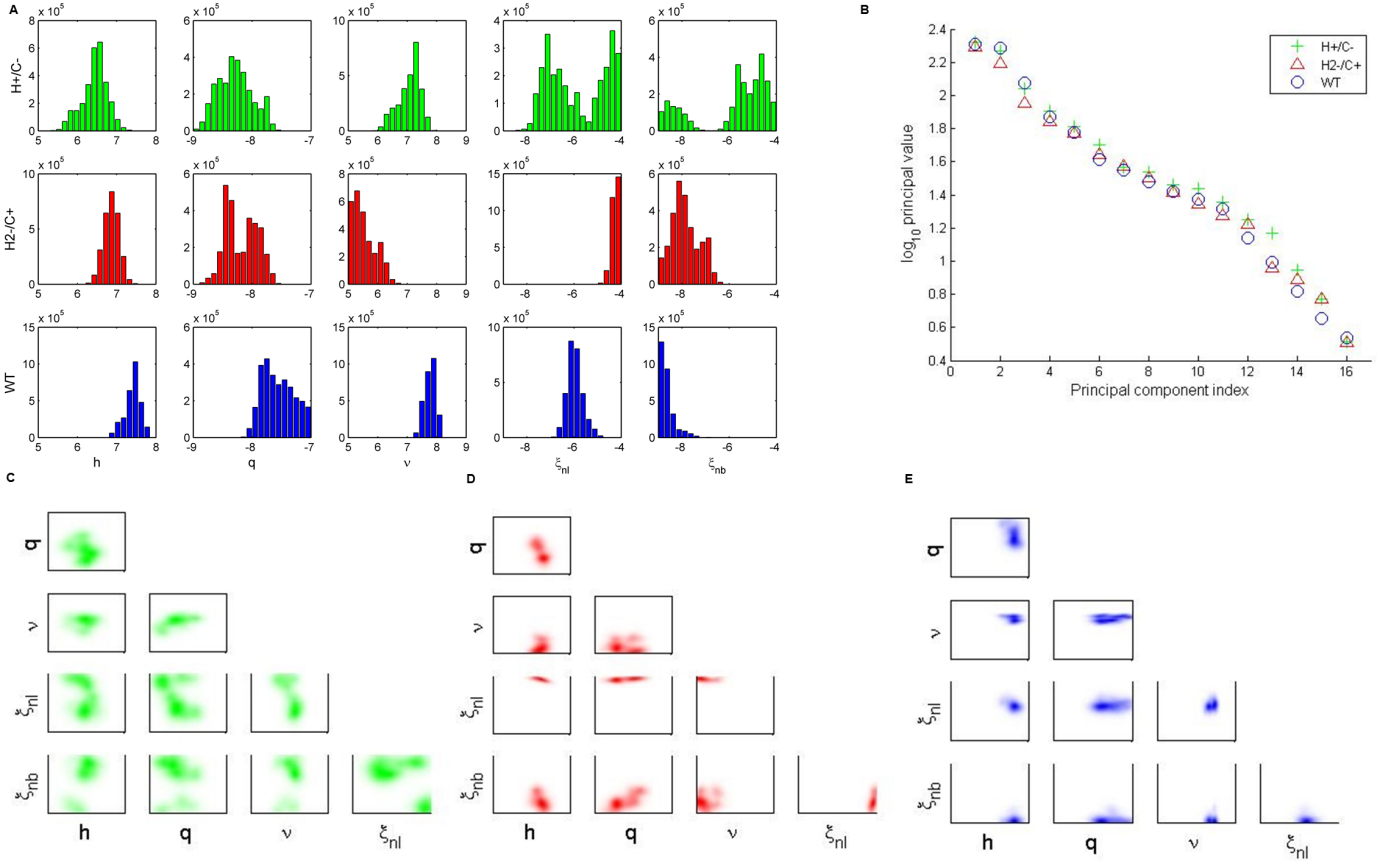
Analysis of bacteria-dependent parameters in the pneumolysin activity study. (A) One-dimensional parameter distributions of bacteria-dependent parameters: *h*, *q*, *ν*, *ξ*_*nl*_, *ξ*_*nb*_. The top row (green) shows ensembles for H+/C- bacteria. The middle row (red) shows ensembles for H2-/C+ bacteria. The bottom row (blue) shows ensembles for wild-type (WT) bacteria. (B) Principal values computed from singular-value decomposition of ensembles for each bacterial strain. (C-E) Two-dimensional parameter correlations for H+/C- (C) H2-/C+ (D) and wild-type (E) bacteria.

We next explore differences in the posterior distributions of the bacteria-strain-dependent parameters (Fig 2A). H+/C- bacteria distributions (Fig 2A, top row) show a bimodal response of the phagocytes, and the pairwise parameter correlations in Fig 2C indicate these parameters are inversely correlated. Thus, when lung phagocytosis rates (*ξ*_*nl*_) are high, blood phagocytosis (*ξ*_*nb*_) tends to be less effective, and vice versa. Since this strain lacks complement activation, we would expect a generally low level of phagocytic activity. Interestingly, even though this strain has full hemolytic activity, the distribution of the damage production rate *q* exists in the lower half of its bounds. This likely occurs because lung bacteria levels remain high throughout the full course of the infection, and thus the values for *q* do not need to be exceedingly high in order to produce movement into the blood compartment.

H2-/C+ bacteria distributions (Fig 2A, middle row) have full complement-activating capability, so they show relatively high levels of phagocytic activation (*h*) and response (*ξ*_*nl*_). Nonspecific clearance (*ν*) tends to be low for this strain, allowing the bacteria to persist in the lungs at a constant level for the first 8 hours post-infection. The lack of hemolytic activity in this bacterial strain is associated with a low level of damage production *q*. In contrast, wild-type bacteria distributions (Fig 2A, bottom row) exhibit high levels of damage production, phagocytic activation, and nonspecific clearance. The pneumolysin of this strain possesses its full hemolytic and complement-activating activity. Clearance of the wild-type bacteria in the blood is low, as the bacteria reach the blood and remain at high levels after about 12 hours post-infection.

We next study the eigenvalues of the system through singular-value decomposition (Fig 2B). Since we see no large difference in the singular values associated with the final two principal components, we conclude that a principal component analysis would not be beneficial to this study, as there is no clear stiff direction in parameter space. Instead, we present two-dimensional parameter correlations for the strain-dependent parameters for each bacteria strain (Fig 2C–2E). Fig 2C more clearly shows the bimodality exhibited by the H+/C- ensemble. The ensembles for H2-/C+ (Fig 2D) and wild-type (Fig 2E) show few correlations of interest, as these distributions tend to be tighter than those of the H+/C- ensemble.

### Neuraminidase study

In the study of Manco *et al*. [23], mice were first infected intranasally with 10^7^°CFU of either wild-type, NanA^−^, or NanB^−^ D39 pneumococcus. NanA^−^ bacteria are cleared from the lungs by 12 hours post-infection, while NanB^−^ bacteria persist for up to 48 hours post-infection but are eventually cleared by the immune system. Wild-type bacteria overwhelm the immune system and cause death about 24 hours post-infection. We select the following parameters to explain the behavior of neuraminidase in the intranasal infection: *q*, the rate of increase of damage to epithelium; *ξ*_*nl*_, the rate of intrapulmonary phagocytosis; and *ν*, the nonspecific clearance of bacteria in the lungs. Changes in *q* and *ν* would represent a decreased ability of the bacteria to bind to the epithelium in the absence of neuraminidase, and some studies also suggest neuraminidase can stimulate resistance to opsonization by neutrophils [28], which would impact the value of *ξ*_*nl*_. We hypothesize that the wild-type bacteria should cause the most damage to the epithelium, as this strain has full ability to bind to the epithelium. We would expect higher rates of clearance and lower damage creation from the mutant strains.

Following the intranasal infection, neither NanA^−^ nor NanB^−^ bacteria were able to be isolated from the blood [23]. To better explore the role of neuraminidase in the blood vessels, Manco *et al*. also infected MF1 mice intravenously with 10^5^ CFU of one of these bacterial strains. Again, the neuraminidase-deficient bacteria are unable to cause a serious infection, as they are cleared from the blood within 2 days post-infection. Wild-type bacteria, however, are again able to cause serious bacteremia and therefore lead to morbidity around 2 days post-infection. We select *a*, the damage-independent rate of bacterial migration from blood to tissue, and *ξ*_*nb*_, the rate of extrapulmonary phagocytosis, to explain the results of the intravenous infection experiment. Neutrophil opsonization would again be limited by the presence of neuraminidase in the wild-type bacteria, and neuraminidase-deficient bacteria would not be able to migrate between compartments easily, as they lack a basic component of adhesion to the epithelial wall. We fit both the intranasal and intravenous data simultaneously for all three strains of bacteria. For the intranasal case, we use an initial condition of 10^7^ CFU for P_L_ and 0 CFU for P_B_, and for the intravenous case, we use an initial condition of 0 CFU for P_L_ and 10^5^ CFU for P_B_, consistent with experimental conditions. The model is fit to data for both cases simultaneously, thus generating only one set of parameter distributions for each strain of mouse in the neuraminidase study.

**Fig 3:**
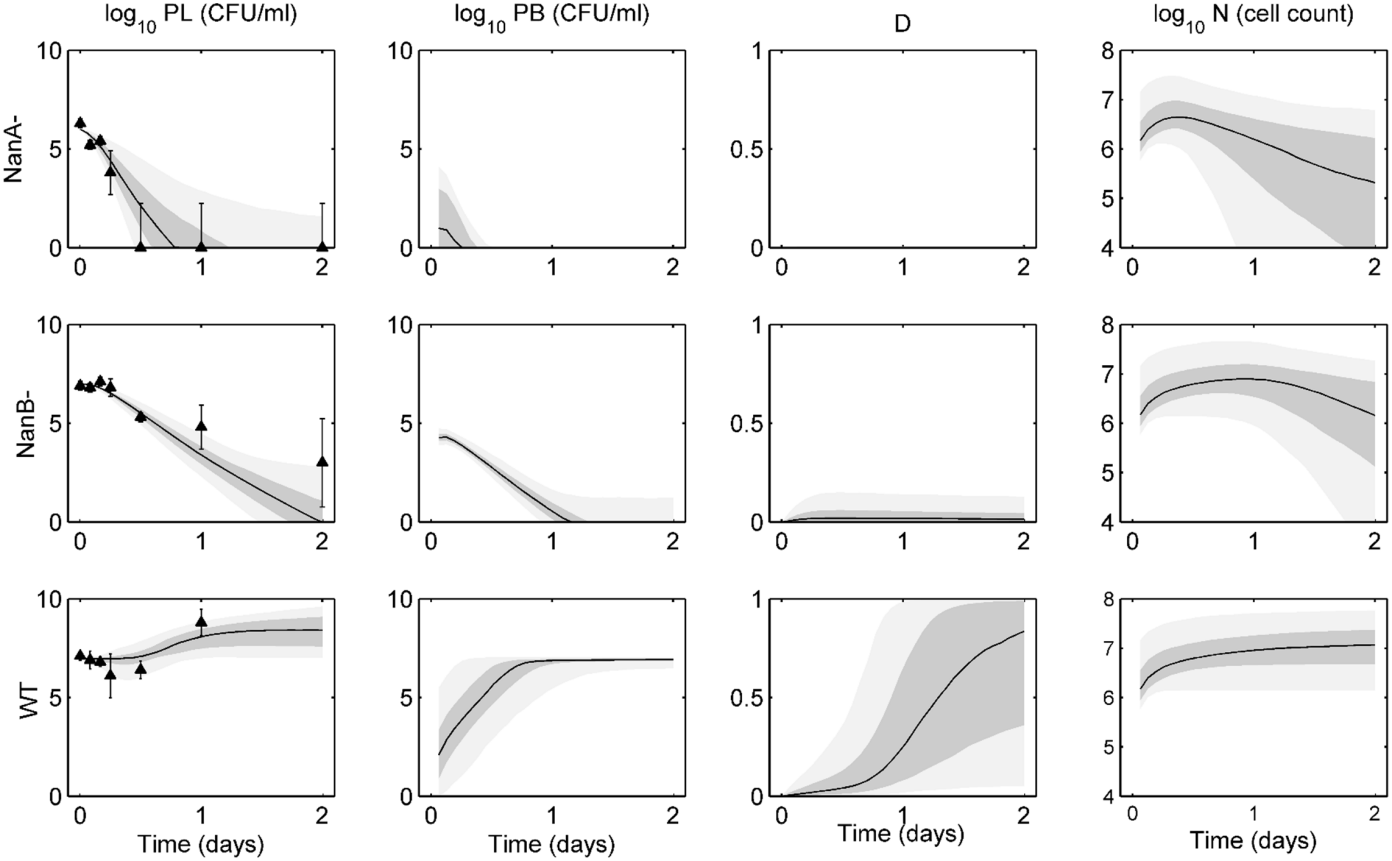
Ensemble trajectories of neuraminidase study with intranasal infection data. Ensemble fits of each strain for lung pathogen (*P*_*L*_), blood pathogen (*P*_*B*_), epithelial damage (*D*), and activated phagocytic cells (*N*). The black line represents the median trajectory, the inner dark gray area represents the 25^th^ to 75^th^ quantiles of trajectories, and the outer light gray envelope represents 90% of the trajectories (5^th^ to 95^th^ quantiles). Data points with standard deviations are represented by the black triangles with error bars. Data were taken at 0, 2, 4, 6, 12, 24, and 48 hours post-infection with five mice in each group. Trajectories are simulated over two days, with infection occurring on day 0 in the lungs. The top row shows ensembles for NanA^−^ bacteria, the middle row shows ensembles for NanB^−^ bacteria, and the bottom row shows ensembles for the wild-type (WT) bacteria.

Fig 3 shows the ensemble fits to intranasal infection data for wild-type, NanA^−^, and NanB^−^ D39 bacteria. NanA^−^ bacteria are unable to adequately bind to the epithelium, and they are cleared from the lungs within 12 hours. NanA^−^ bacteria are unable to cause any appreciable damage or sustain a population in the blood for more than a few hours in our predicted trajectories. Experiments verified that these bacteria were not detected in the blood at any point in the experiments. NanB^−^ bacteria can persist in the lungs longer than NanA^−^, but these bacteria will eventually clear as well. While our ensembles show some presence of bacteria in the blood, these bacteria are cleared within about one day, thus not causing severe bacteremia, again aligning with the findings of the authors [23]. The wild-type bacteria are highly virulent, causing sepsis and eventual death to the mice about 1 day post-infection. Our ensembles match the lung data well and show a quick rise in blood bacteria levels as well as epithelial damage. Though the activated phagocytic cell population is highest in the simulated wild-type bacteria ensemble, these cells are unable to contain the bacterial population in either compartment.

**Fig 4:**
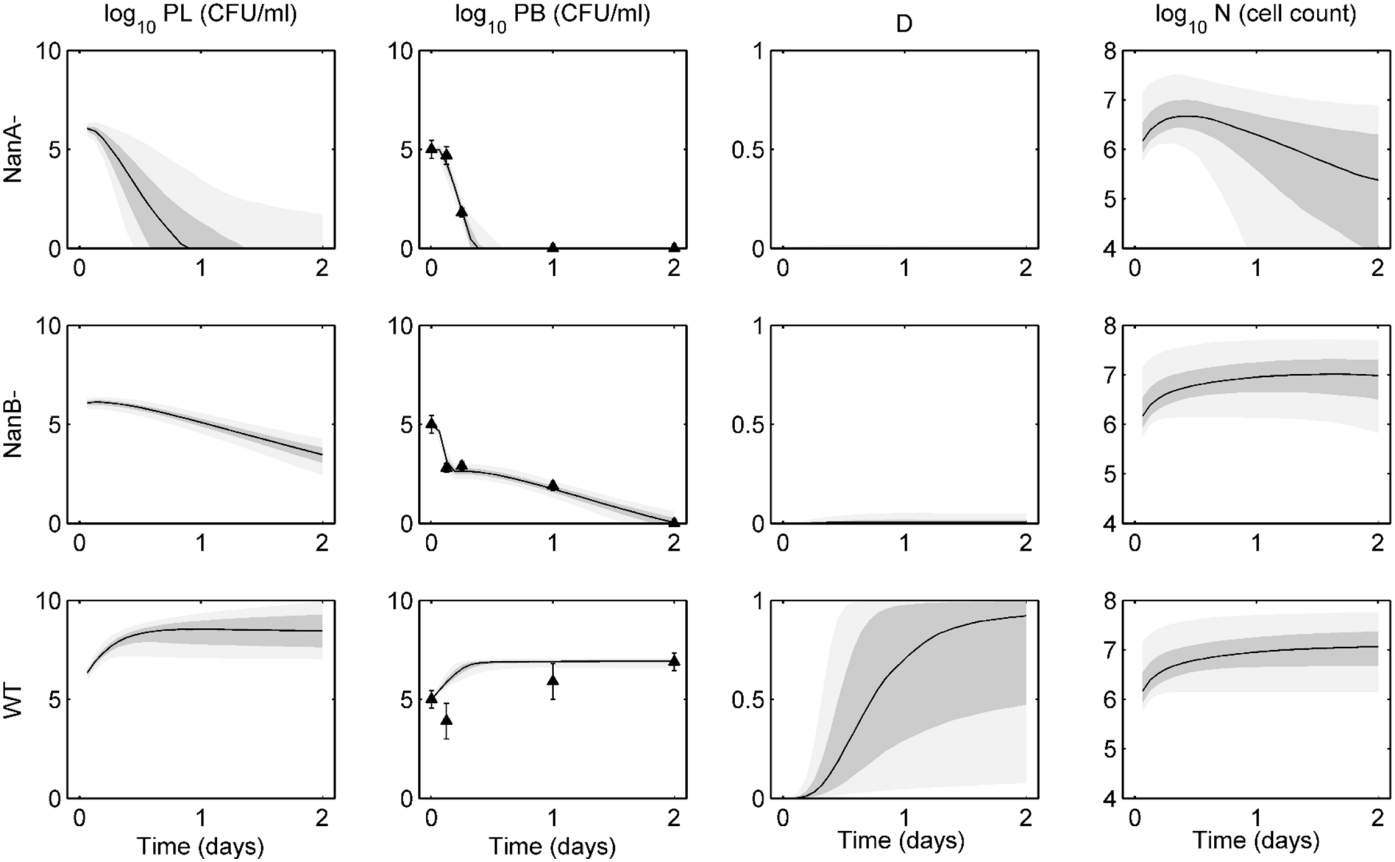
Ensemble trajectories of neuraminidase study with intravenous infection data. Ensemble fits of each strain for lung pathogen (*P*_*L*_), blood pathogen (*P*_*b*_), epithelial damage (*D*), and activated phagocytic cells (*N*). The black line represents the median trajectory, the inner dark gray area represents the 25^th^ to 75^th^ quantiles of trajectories, and the outer light gray envelope represents 90% of the trajectories (5^th^ to 95^th^ quantiles). Data points with standard deviations are represented by the black triangles with error bars. Data were taken at 0, 3, 6, 24, and 48 hours post-infection with five mice in each group. Trajectories are simulated over two days, with infection occurring on day 0 in the blood. The top row shows ensembles for NanA^−^ bacteria, the middle row shows ensembles for NanB^−^ bacteria, and the bottom row shows ensembles for the wild-type (WT) bacteria.

Fig 4 demonstrates the ensemble solutions for the intravenous infection experiments. Bacteria are introduced into the blood at day 0 at an initial level of 10^5^ CFU. NanA^−^ bacteria are cleared from the blood within about 12 hours post-infection, and while they are able to reach the lungs relatively quickly, they are cleared from the tissue quickly as well. NanB^−^ bacteria show an initial steep drop in blood levels as they move into the lungs. These bacteria are not fully cleared from the blood until about 2 days post-infection. While the lung bacteria levels have not been eliminated at this point, all trajectories will eventually lead to total clearance of bacteria from both compartments. Again, the wild-type bacteria are the most virulent in these experiments. These are the only bacteria able to cause significant damage, and as such the bacteria levels in both compartments rise over the first 12 hours until they hit a carrying capacity in the blood and cause morbidity of the host.

**Fig 5:**
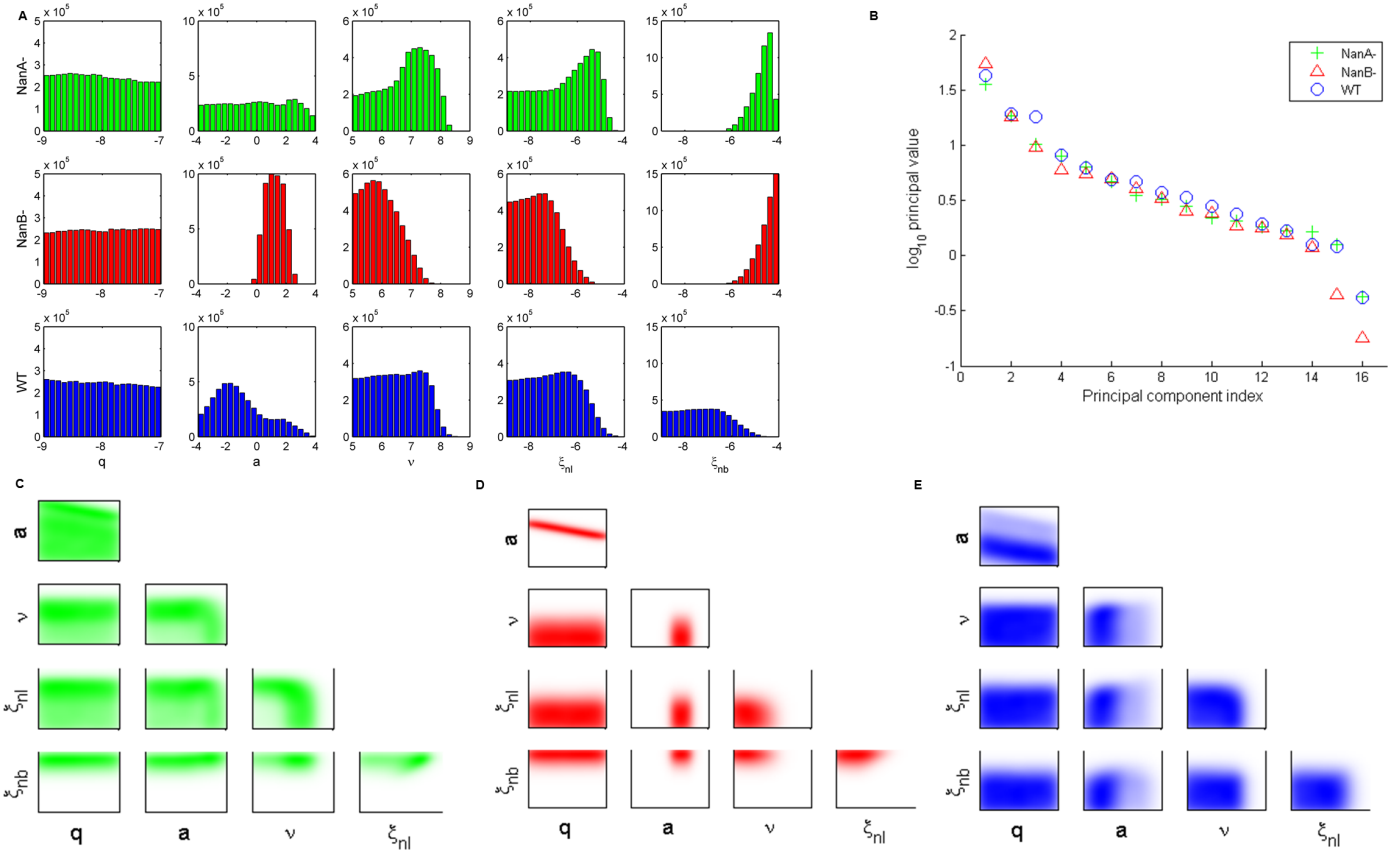
Analysis of bacteria-dependent parameters in the neuraminidase study. (A) One-dimensional parameter distributions of bacteria-dependent parameters: *q*, *a*, *ν*, *ξ*_*nl*_, *ξ*_*nb*_. The top row (green) shows ensembles for NanA^−^ bacteria. The middle row (red) shows ensembles for NanB^−^ bacteria. The bottom row (blue) shows ensembles for wild-type (WT) bacteria. (B) Principal values computed from singular-value decomposition of ensembles for each bacterial strain. (C-E) Two-dimensional parameter correlations for NanA^−^ (C) NanB^−^ (D) and wild-type (E) bacteria.

Distributions of our bacteria-dependent parameters show marked differences across these three strains (Fig 5A). NanA^−^ bacteria are essentially insensitive to *q* and *a*, as the bacteria are unable to maintain their population for more than a few hours in either the intranasal or the intravenous experiments. Clearance rates of the NanA^−^ bacteria both by nonspecific means (*ν*) and by phagocytic cells (*ξ*_*nl*_, *ξ*_*nb*_) tend towards the upper end of the spectrum, meaning these bacteria are easily cleared by the immune system. This result is further verified by the evidence that NanA prevents opsonization by neutrophils [28].

NanB^−^ bacteria show the most sensitivity to *a*, the damage-independent movement of bacteria from blood to lungs, as this distribution is the most narrow. Pulmonary clearance of the NanB^−^ bacteria (*ν*, *ξ*_*nl*_) tends to be lower than that of the NanA^−^ bacteria. It is unclear whether NanB has the same effect on opsonization as NanA; further experiments on the interactions of NanB and neutrophils are needed in order to verify this prediction biologically. The clearance of NanB^−^ bacteria in the blood is generally very high, explaining the fast initial drop in blood levels in the intravenous infection data.

The distributions for the wild-type bacteria differ most from the neuraminidase-deficient bacteria in the values of *a* and *ξ*_*nb*_, both of which are much lower than the distributions seen in the other two strains. Both of these results align with our initial hypotheses; the presence of NanA allows the wild-type bacteria additional resistance to phagocytosis, and the ability to bind the epithelium and cause excess damage means the bacteria require less damage-independent motion to overwhelm the lung and blood compartments. Interestingly, all three strains show an insensitivity to *q*, despite the known increased ability of the wild-type bacteria to adhere to the epithelium and create damage. The effect of this phenomenon is absorbed in the parameter *a*; higher values of *a* imply lower levels of damage created.

The principal values of the system again show no significant difference between the final two components (Fig 5B), so a principal component analysis would not provide interpretable results. The two-dimensional parameter correlations (Fig 5C–5E) show a few noteworthy patterns. NanA^−^ bacteria exhibit a switching behavior with *ν*, *a*, and *ξ*_*nl*_. When *ν* is high, then *a* and *ξ*_*nl*_ are low, and vice versa. In addition, both NanB^−^ and wild-type bacteria show a strong negative correlation between *q* and *a*.

### Serotype study

In the study of McCluskey *et al*. [26], mice were given wild-type strains of either D39 (serotype 2), 0100993 (serotype 3), or TIGR4 (serotype 4) pneumococcus. These strains differ primarily in the serotypes’ capsule thicknesses and virulence. In this study, we selected 6 parameters as bacteria-strain-dependent: *h*, *q*, *a*, *ν*, *ξ*_*nl*_ and *ξ*_*nb*_, since these parameters control the degree to which bacteria can move between lung tissue and blood (*q* and *a*), as well as the degree to which the host is able to fight these particular strains of pneumococcus (*h*, *ν*, *ξ*_*nl*_, *ξ*_*nb*_).

**Fig 6:**
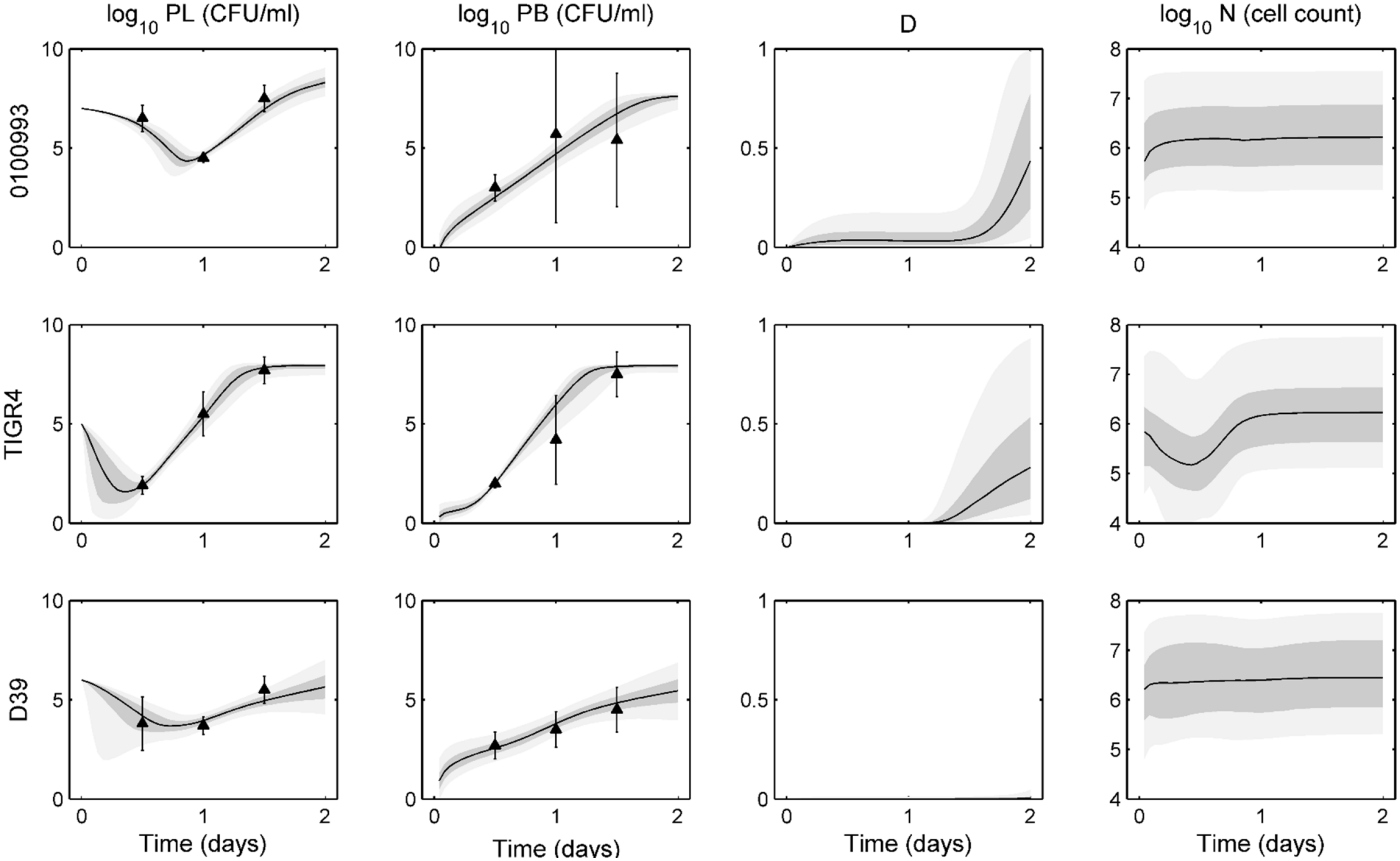
Ensemble trajectories of serotype study. Ensemble fits of each strain for lung pathogen (*P*_*L*_), blood pathogen (*P*_*B*_), epithelial damage (*D*), and activated phagocytic cells (*N*). The black line represents the median trajectory, the inner dark gray area represents the 25^th^ to 75^th^ quantiles of trajectories, and the outer light gray envelope represents 90% of the trajectories (5^th^ to 95^th^ quantiles). Data points with standard deviations are represented by the black triangles with error bars. Data were taken at 12, 24, and 48 hours post-infection with three mice in each group. Trajectories are simulated over two days, with infection occurring on day 0 in the blood. The top row shows ensembles for 0100993 bacteria, the middle row shows ensembles for TIGR4 bacteria, and the bottom row shows ensembles for the D39 bacteria.

Fig 6 shows the ensemble trajectories fit to data for D39, 0100993, and TIGR4 bacteria up to 48 hours post-infection. Trajectories generally fit data tightly, with most variation in predicted trajectories occurring in *D* and *N*. TIGR4 tend to create the most damage, while very little damage is seen for the D39 ensemble. Each strain varies significantly in the first 12 hours, represented by the first data point. 0100993 bacteria remain relatively high, exhibiting little nonspecific clearance. TIGR4 and D39 bacteria levels in the lung decrease by several orders of magnitude during the first 12 hours, showing both a greater susceptibility to this initial clearance and faster movement into the bloodstream.

**Fig 7:**
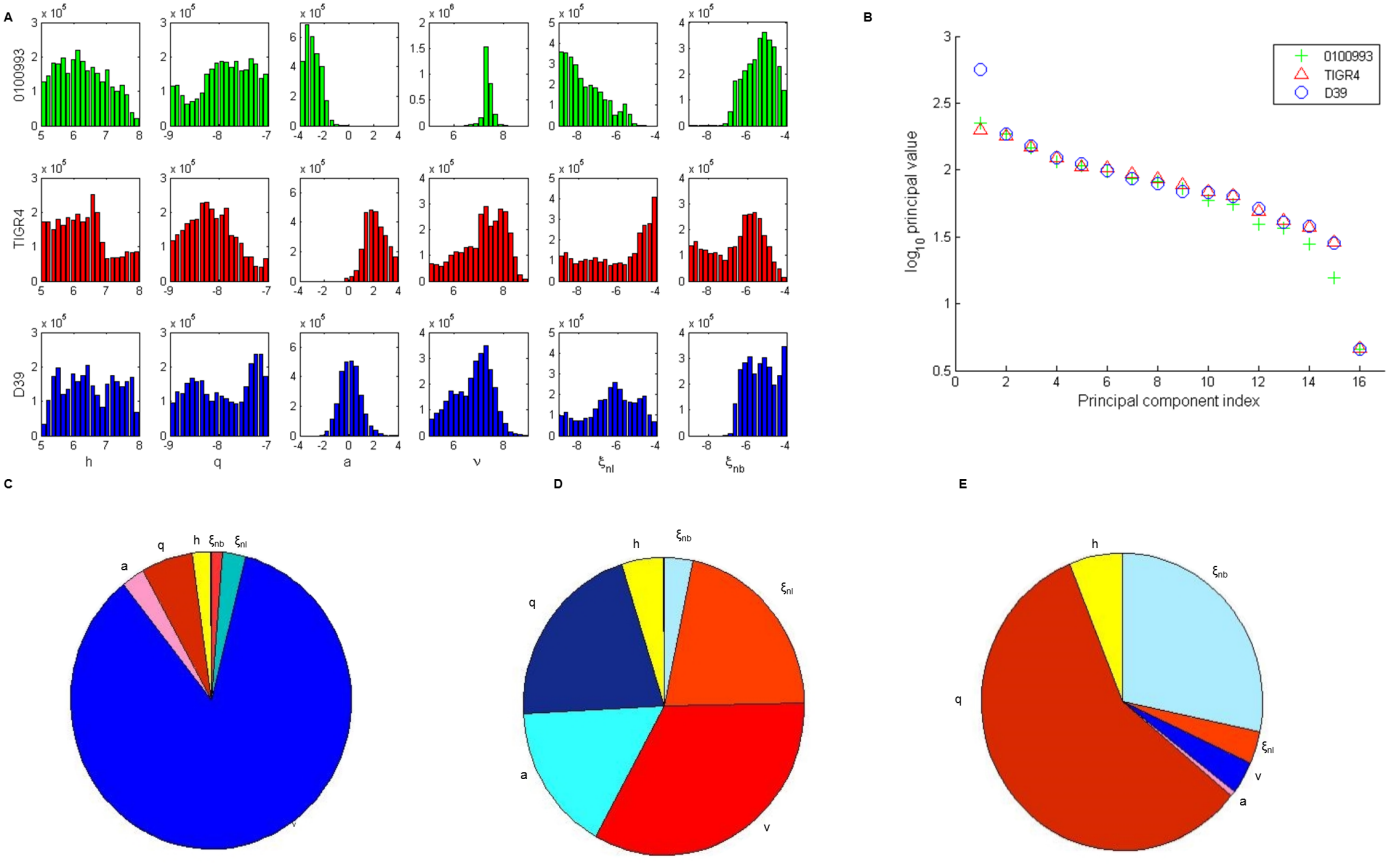
Analysis of bacteria-dependent parameters in the sesrotype study. (A) One-dimensional parameter distributions of bacteria-dependent parameters: *h*, *q*, *a*, *ν*, *ξ*_*nl*_, *ξ*_*nb*_. The top row (green) shows ensembles for 0100993 bacteria. The middle row (red) shows ensembles for TIGR4 bacteria. The bottom row (blue) shows ensembles for D39) bacteria. (B) Principal values computed from singular-value decomposition of ensembles for each bacterial strain. (C-E) Composition of the last principal component for 0100993 (C) TIGR4 (D) and wild-type (E) bacteria. Warm colors represent a negative contribution to sensitivity, and cool colors represent a positive contribution.

Fig 7A shows the distributions of bacteria-strain-dependent parameters for each of the three serotypes. The largest disparities between strains exist in distributions for *a*, *ξ*_*nl*_, *ξ*_*nb*_ and *ν* populations. 0100993 bacteria tend to have a low value for *a*, the damage-independent movement of bacteria from the bloodstream to the tissue. Since these serotype 3 bacteria typically remain higher in the lung tissue than in the blood, we would expect the effect of this motion to be minimal. In contrast, TIGR4 bacteria tend to have a high *a* value, as these bacteria readily cause sepsis. D39, known to cause both severe pneumonia and sepsis in MF1 mice, have *a* values concentrated between these two extremes.

Each serotype also differs in its resistance to clearance by both mucosal immunity and phagocytic activity. The distribution for D39 *ν* aligns with the MF1 output from our previous work [24]. Higher values of *ν* are evident in 0100993 and TIGR4 bacteria. Phagocytosis rates (*ξ*_*nl*_ and *ξ*_*nb*_) also vary between these serotypes. Phagocytosis in the tissue tends to be low for the 0100993 bacteria, high for TIGR4, and D39 lies somewhere in the middle. Extrapulmonary phagocytosis is high in both 0100993 and D39 and slightly lower for TIGR4.

We perform singular value decomposition on the ensemble, and from the output determine the makeup of each principal component and eigenvalues associated with each. The steep drop in the magnitude of the eigenvalue associated with the final principal component suggests that the composition of the final principal component explains most of the sensitivity of the model (Fig 7B). We study the makeup of this vector to determine which parameters contribute most to this sensitivity (Fig 7C). The relative contribution of each element to the vector is represented in a pie chart, and the color of each piece denotes whether the parameter has a positive (cool colors) or negative (warm colors) contribution.

Strain 0100993 is most sensitive to *ν*. Serotype 3 strains tend to stay in the lungs to cause severe pneumonia [29], so a sensitivity to clearance in the tissue is expected. The TIGR4 ensemble is most sensitive to *ν*, *ξ*_*nb*_, *q* and *a*. TIGR4 bacteria are known to cause bacteremia and sepsis very quickly in mice, and eventually progress to cross the blood-brain barrier, leading to meningitis [29]. These sensitive parameters control the ability of the bacteria to proliferate in the lungs, cause epithelial damage, and move between the lung and blood compartments. Lastly, the D39 ensemble is most sensitive to *q*. D39 is known to be highly virulent to mice, often leading to both sepsis and severe pneumonia [29,30]. Since bacterial replication in lung tissue is highly dependent on the ability of the bacteria to move to the blood and evade phagocytosis, we would expect the rate of damage creation to be critical to explain the data.

## Discussion

In the present study we further develop a recently proposed ODE model of intrahost immune response to pneumococcal pneumonia [24]. We demonstrate the selection of a parsimonious subset of parameters that can define primary differences between bacterial strains. We here show that the model is capable of capturing differences not only across murine strains (as described in [24]), but also across bacterial strains. Our model identifies differences in immune response to infection by bacteria missing a portion of a protein (pneumolysin activity study), a whole protein (neuraminidase study), or entirely different serotypes (serotype study). The model can also describe initial decay and subsequent dramatic rise in the number of pneumococci in the lungs, which has been observed experimentally as coordinated with a similar rise in the blood.

While a different subset of five or six parameters was identified as bacteria-dependent in each study, four parameters were consistently bacteria-dependent in all of our three studies: *ν*, *q*, *ξ*_*nl*_ and *ξ*_*nb*_. The parameter *ν* incorporates nonspecific clearance mechanisms such as mucociliary clearance, alveolar macrophage activity, defensins and other proteins active in mucosal immunity. The pneumolysin study demonstrated differences in *ν* for all three bacterial strains, with high *ν* values seen in the wild-type bacteria ensembles and low *ν* values in the H2-/C+ ensembles. Absence of hemolytic activity in the H2-/C+ bacteria could be responsible for a decreased activation of the alveolar macrophages, thereby decreasing the overall rate of clearance by *ν*. In the neuraminidase study, *ν* tended to be lowest for NanB^−^ bacteria. These bacteria have not been studied extensively, so reasons for this difference are unclear.

In the serotype study, *ν* stood out as a highly sensitive parameter for both TIGR4 and 0100993 bacteria. TIGR4 bacteria are highly virulent, so likely the host must have strong nonspecific defenses to control TIGR4 levels from the beginning of the infection. Another virulence factor impacting the nonspecific clearance is choline-binding protein. Choline-binding proteins allow bacteria to anchor themselves to the epithelial surface, increasing their ability to avoid non-specific clearance mechanisms. Brooks-Walter *et al*. showed that about 25% of pneumococcal strains do not express choline-binding protein A (CbpA, also known as PspC or SpsA), which can limit virulence [31]. It has been shown that another serotype 3 strain, A66.1, does not express CbpA, so it is possible that our serotype 3 strain, 0100993, also does not. This would explain the need for such a high inoculum to generate survival rates similar to those seen in the other infections; an inoculum of 10^7^°CFU of 0100993 was required, compared to only 10^5^ CFU of TIGR4, suggesting decreased virulence in the serotype 3 strain.

The parameter *q*, the rate at which lung bacteria create damage to the epithelium, was found to be strain-dependent in all three studies. While *q* was not an influential parameter in the neuraminidase study, it was crucial to the ensemble fits for the wild-type D39 bacteria in the pneumolysin and serotype studies. D39 is often used in murine models of pneumonia because it is known to be highly virulent to mice, leading to both severe pneumonia and bacteremia. We would therefore expect to see damage creation as a highly important factor in the phenotype associated with D39 infection. The *q* distribution for wild-type D39 tends to be skewed high in the pneumolysin study but exists over the full parameter range in the serotype study. We can see large differences in the D39 bacteria levels between the two studies, possibly due to different laboratory conditions, different protocols used, or different sources of the materials and specimens used in the study. These differences are enough to induce distinct parameter distributions for each wild-type ensemble.

The rates of phagocytic clearance in lung tissue and blood (*ξ*_*nl*_ and *ξ*_*nb*_, respectively) were also found to be bacteria-dependent parameters in each of the three studies. Many virulence factors present on the bacterial surface allow the bacteria to avoid phagocytosis, and since the presence and efficacy of these virulence factors varies across strains, we would expect these parameters to greatly influence the ensembles. In the pneumolysin study, because the biggest variations in the data occur in the first 12 hours, phagocytic cells do not control the major differences in the ensembles; these effects are felt more strongly later in the course of the infection. In the neuraminidase study, wild-type bacteria are not cleared effectively in the blood, while the neuraminidase-deficient strains show very high levels of blood clearance. Neuraminidase A is known to decrease efficacy of neutrophil killing [28], so it is possible neuraminidase B has a similar effect on the host. Our ensembles do not demonstrate such a stark contrast in the intrapulmonary phagocytosis rates, however. A lower intensity of immune response in the lungs is sufficient to clear the bacteria.

We have utilized experimental data for neutrophil levels and for bacterial levels in the lungs and in the blood to calibrate the model. We have not fit the trajectory of the damage variable to any data and therefore this trajectory is a prediction of the model that can be potentially used to validate our results. There are several ways that damage to epithelium can be monitored; one possibility is by means of a biomarker such as decreased lung capacity or decreased epithelial cell cilia [32,33]. The other possibility is to use histological samples to assess the level of epithelial damage. The addition of damage level data to future uses of the model could change the distributions of *q*, possibly making them to be tighter to adhere to a particular range of data.

The ensemble model approach to data analysis can accommodate uncertainty in data, but it does have limitations. Our equation-based model might be considered complex by some researchers (it requires 17 parameters), yet, even so, it greatly simplifies the actual biology of the immune response. For example, parameter *ν* lumps several different mechanisms involved in mucosal immunity, only one type of immune cells is assumed to remove pathogens, and we do not directly account for intercellular signaling. Unfortunately, experimental datasets required to parametrize more complex and biologically accurate models describing these mechanisms currently do not exist. Accordingly, we perceive our contribution as hypothesis-generating and as a basis for guiding future mechanistically-based experimentation. Future iterations of the model could address some of these simplifying assumptions, perhaps providing for a more detailed account of the host immune system. Another important assumption of the model is that, within the lung or blood compartments, the system is well-mixed. In reality, there is a spatial component to bacterial infections that this simple model is unable to capture. Future models may allow for this spatial heterogeneity to be incorporated into the dynamics.

In conclusion, we have presented an important extension of our previously proposed model that shows its utility not only in modeling how different hosts response to the same bacterial infection, but also how identical hosts respond to multiple types of bacterial infections. Our model is able to capture the initial decay followed by quick rise in lung bacterial loads associated with a rise in blood bacterial loads. We have demonstrated how the parameters of our model can be used to analyze and predict the immune responses of the host to each type of bacteria. This work provides a path forward for future work modeling the response to different bacterial strains or adding complexity to the model by incorporating more components of the immune system explicitly in the equations.

## Methods

### Experimental data

The model is calibrated to literature data [23,25,26] for female MF1 mice given an intranasal dose of pneumococcus. The pneumolysin study uses data of Jounblat *et al*., [25], where mice were infected with D39 bacteria to study the impact of pneumolysin on the survival of bacteria in the body. Mice were given a dose of 10^6^ CFU of either wild-type D39 or one of two mutant strains: H+/C- or H2-/C+. H+/C- mutants feature pneumolysin that lacks the complement-activating activity but have no change to the hemolytic activity. In contrast, H2-/C+ mutants have full complement-activating activity but exhibit only 0.02% of normal pore-forming activity. Data are given for bacterial populations in both the lung tissue and blood at 0, 3, 6, 12, 24, and 48 hours post-infection. The neuraminidase study utilizes data from Manco *et al*. [23], where mice were infected with 10^7^°CFU of either wild-type, neuraminidase A deficient (NanA^−^), or neuraminidase B deficient (NanB^−^) D39 pneumococcus. Data are given only for bacterial populations in the lung tissue for 0, 2, 4, 6, 12, 24, and 48 hours post-infection. The serotype study incorporates data from McCluskey *et al*. [26], where mice were given wild-type strains of either 10^6^ CFU of D39, 10^7^ CFU of 0100993, or 10^5^ CFU of TIGR4 pneumococcus. Data are given for bacterial load in lungs and blood at 12, 24, and 36 hours post-infection.

### Mathematical model

In the intranasal infection data, bacteria are introduced intranasally into the lung tissue, where they quickly attach to the epithelium and begin to reproduce. The first line of defense against these bacteria is nonspecific immunity, such as resident alveolar macrophages and mechanical clearance via the mucociliary elevator. Together, these defenses can clear low levels of bacteria without the need for an additional influx of immune cells. If bacteria levels are too high for nonspecific clearance alone, lung epithelial cells will signal an influx of white blood cells, primarily neutrophils [34]. These phagocytes will become activated and move to the site of infected tissue. Though phagocytes will ingest bacteria as they move through the bloodstream to the lungs, the majority of their work will occur in the lung tissue. When bacteria adhere to the epithelium, they cause increased cell death and damage to the epithelium. This damage facilitates the exchange of bacteria between tissue and blood by increasing the permeability of the epithelium [32,33]. Bacteria can also move in a damage-independent motion; bacteria in the blood have a tendency to move back to tissue to colonize [35].

These mechanisms were utilized by Mochan *et al*. [24] in the development of a four equation ODE model used there and also in this study. The model predicts the time-dependent trajectories for four variables: bacterial load in lung tissue (*P*_*L*_), bacterial load in blood (*P*_*B*_), damage to the epithelial barrier between lungs and blood (*D*), and the activated phagocyte population (*N*):

$$\frac{dP_{L}}{dt}=k_{l}P_{L}-\frac{\nu P_{L}}{\mu +P_{L}}-\frac{\xi_{nl}NP_{L}}{1+\xi_{2}P_{L}}+f\left[(bD+a)P_{B}-bDP_{L}\right]$$ (1)

$$\frac{dP_{B}}{dt}=k_{b}P_{B}\left(\frac{P_{B}-\varepsilon }{P_{B}+\varepsilon }\right)\left(1-\frac{P_{B}}{K}\right)-\frac{\xi_{nb}NP_{B}}{1+\xi_{2}P_{B}}-(bD+a)P_{B}+bDP_{L}$$ (2)

$$\frac{dD}{dt}=qP_{L}(1-D)-cD$$ (3)

$$\frac{dN}{dt}=\left(-N+\frac{hP_{L}}{n_{h}+P_{L}}\right)\left(\frac{1}{\tau_{N}}\right)$$ (4)

In the model, bacteria in the lungs grow at a linear rate with constant *k*_*l*_, and are removed by (i) mechanical clearance via cilia and mucus and phagocytosis by alveolar macrophages, modeled with rate constant *ν* and saturation constant *μ*, and by (ii) neutralization by phagocytes (*N*) with rate constant *ξ*_*nl*_ and saturation constant *ξ*_2_. Movement of bacteria between lungs and blood is described by the last two terms, where *f* describes the volumetric difference between the blood and lung compartments, *a* describes the damage-independent rate of movement of the blood bacteria into the lung compartment, and *b* is the permeability of the membrane to the bacteria, describing the damage-dependent movement. Blood pathogen (*P*_*B*_) grows logistically with rate constant *k*_*b*_, carrying capacity *K*, and the Allee threshold *ε* that represents the extinction limit. Blood pathogens are removed by phagocytes (*N*) in the blood with rate constant *ξ*_*nb*_ and saturation constant *ξ*_2_. Like the previous equation, the final terms describe damage-dependent and damage-independent motion of bacteria between the two compartments. Damage (*D*) increases at a rate proportional to the amount of bacteria present in the lung, with rate constant *q*. The body is able to repair damage at a linear rate with constant *c*. The damage varies only between 0 and 1. The dynamics of phagocytes (*N*) occurs on time-scale *τ*_*N*_. Activation is proportional to the population of lung pathogen. The activation and influx of the phagocytes is proportional to the lung pathogen level with rate constant *h*, and saturates with a half-maximum value at *n*_*h*_.

### Markov Chain Monte Carlo simulations

As in our previous work, we use Bayesian inference to estimate the posterior distributions of each of the parameters of our model. For each of the three studies conducted, we have *n* bacteria-strain-dependent parameters defined and 17−*n* bacteria-strain-independent parameters. (See Table 1 for a full list of strain-dependent parameters assigned in each study.) Parameter bounds are identical to those in our previous work, except for the bounds on parameters *a* and *K*. Bounds on *a* have been widened to include values between 10^−4^−10^4^. To reduce dimensionality, *K*, the carrying capacity of the bacteria, was kept at a constant 10^8^ CFU in all iterations of all studies. In our previous work, *K* was allowed to vary between 10^7^ and 10^8^ CFU. Here, we set *K* to its upper bound of 10^8^ CFU so that it would be unlikely to hinder any of our fits to these bacterial strains, as some of the lung bacteria data used in this study reach a steady state value close to 10^8^ CFU. Table 2 shows the full ranges for each of the parameters in the model.

**Table 2:** Full list of parameters of the model, their biological interpretations, and the ranges over which they are varied in the ensemble.

Parameter	Meaning	Units	Bounds
*τ*_*N*_	Time to activation of phagocytes	hours	0.01–10
*n*_*h*_	Steady-state value of phagocyte population	cells	10^2^–10^4^
*μ*	Michaelis-Menten rate of nonspecific immunity	CFU/ml	10^5^–10^7^
*ξ*_2_	Inhibition of phagocyte phagocytosis	ml/CFU	10^−13^–10^−11^
*h*	Activation of phagocytes due to *P*_*L*_ population	cells/CFU/ml	10^5^–10^8^
*K*	Carrying capacity of blood pathogen	CFU/ml	10^7^–10^8^
*k*_*l*_	Growth rate of pathogen in lung tissue	hour^-1^	0.1–5
*k*_*b*_	Growth rate of pathogen in blood	hour^-1^	20–40
*q*	Rate of increase of damage due to *P*_*L*_	hour^-1^	10^−9^–10^−7^
*ε*	Threshold value of *P*_*B*_	CFU/ml	1–10
*a*	Directionality of movement from *P*_*B*_ to *P*_*L*_	hour^-1^	10^−4^–10^4^
*f*	Volumetric ratio	dimensionless	10–50
*b*	Effect of *D* on movement rates	hour^-1^	0.01–10
*ν*	Rate of clearance of *P*_*L*_ by nonspecific immunity	CFU/ml/hour	10^4^–10^9^
*ξ*_*nl*_	Rate of phagocyte phagocytosis in lung tissue	cell^-1^ hour^-1^	10^−9^–10^−4^
*ξ*_*nb*_	Rate of phagocyte phagocytosis in blood	cell^-1^ hour^-1^	10^−9^–10^−4^
*c*	Rate of repair of damage	hour^-1^	0.01–1

At each iteration of the algorithm, parameter vector **p** consists of 3*n* strain-dependent parameter values (one set of *n* parameters for each type of bacteria used in the study) and 17−*n* strain-independent parameter values. Bounds on the parameters were defined in biological ranges where literature values could be determined and estimated for the other parameters.

To ensure the parameters chosen as strain-dependent are the most sensitive in each study, we first sampled a posterior distribution in which we allowed all 17 parameters to vary between each strain. We observed which parameters tended to localize to different areas of parameter space and identify those as the most sensitive parameters. We then chose those parameters strain-dependent [24] and resampled the posterior distribution.

Given the high dimensional space in which parameters must be sampled, we use a parallel tempering algorithm [36–38]. We generated four million parameter sets for each of the three studies. Convergence of the chains was verified with the Gelman-Rubin test [39] and the Geweke test [40].

## References

[1] World Health Organization. Pneumonia fact sheet. In: Media Centre. 2013.

[2] Kadioglu A, Taylor S, Iannelli F, Mitchell TJ, Andrew PW, Pozzi G. Upper and Lower Respiratory Tract Infection by Streptococcus pneumoniae Is Affected by Pneumolysin Deficiency and Differences in Capsule Type. Infect Immun. 2002;70: 2886–2890. 12010976 10.1128/IAI.70.6.2886-2890.2002 PMC128015

[3] Kelly T, Dillard JP, Yother J. Effect of genetic switching of capsular type on virulence of Streptococcus pneumoniae. Infect Immun. 1994;62: 1813–9. Available: http://www.pubmedcentral.nih.gov/articlerender.fcgi?artid=186414&tool=pmcentrez&rendertype=abstract 8168944 10.1128/iai.62.5.1813-1819.1994 PMC186414

[4] Mohler J, Azoulay-Dupuis E, Amory-Rivier C, Mazoit JX, Bédos JPP, Rieux V, et al Streptococcus pneumoniae strain-dependent lung inflammatory responses in a murine model of pneumococcal pneumonia. Intensive Care Med. 2003;29: 808–16. 12665994 10.1007/s00134-003-1699-x

[5] Avery OT, Dubos R. The protective action of a specific enzyme against type III pneumococcus infection in mice. J Exp Med. 1931;54: 73–89. 19869903 10.1084/jem.54.1.73 PMC2132045

[6] Watson DA, Musher DM. Interruption of capsule production in Streptococcus pneumoniae serotype 3 by insertion of transposon Tn916. Infect Immun. 1990;17: 913–924. 10.1128/iai.58.9.3135-3138.1990 PMC313622 2167295

[7] Lee C-J, Banks SD, Lee JP. Virulence, immunity, and vaccine related to Streptococcus pneumoniae. Crit Rev Immunol. 1991;18: 89–114. 10.3109/10408419109113510 1930677

[8] Bruyn G a, Zegers BJ, van Furth R. Mechanisms of host defense against infection with Streptococcus pneumoniae. Clin Infect Dis. 1992;14: 251–62. Available: http://www.ncbi.nlm.nih.gov/pubmed/1571441 1571441 10.1093/clinids/14.1.251

[9] Todar K. Todar’s Online Textbook of Bacteriology. 2008.

[10] Jay SJ, Johanson WG, Pierce a K, Reisch JS. Determinants of lung bacterial clearance in normal mice. J Clin Invest. 1976;57: 811–7. 10.1172/JCI108356 7575 PMC436723

[11] Fine DP. Pneumococcal type-associated variability in alternate complement pathway activation. Infect Immun. 1975;12: 772–8. Available: http://www.pubmedcentral.nih.gov/articlerender.fcgi?artid=415355&tool=pmcentrez&rendertype=abstract 329 10.1128/iai.12.4.772-778.1975 PMC415355

[12] Giebink GS, Verhoef J, Peterson PK, Quie PG. Opsonic requirements for phagocytosis of Streptococcus pneumoniae types VI, XVIII, XXIII, and XXV. Infect Immun. 1977;18: 291–7. Available: http://www.pubmedcentral.nih.gov/articlerender.fcgi?artid=421229&tool=pmcentrez&rendertype=abstract 21849 10.1128/iai.18.2.291-297.1977 PMC421229

[13] Hostetter MK. Serotypic variations among virulent pneumococci in deposition and degradation of covalently bound C3b: implications for phagocytosis and antibody production. J Infect Dis. 1986;153: 682–93. Available: http://www.ncbi.nlm.nih.gov/pubmed/3950449 3950449 10.1093/infdis/153.4.682

[14] Chudwin DS, Artrip SG, Korenblit a, Schiffman G, Rao S. Correlation of serum opsonins with in vitro phagocytosis of Streptococcus pneumoniae. Infect Immun. 1985;50: 213–7. Available: http://www.pubmedcentral.nih.gov/articlerender.fcgi?artid=262158&tool=pmcentrez&rendertype=abstract 4044034 10.1128/iai.50.1.213-217.1985 PMC262158

[15] Kalin M. Pneumococcal serotypes and their clinical relevance. Thorax. 1998;53: 159–62. Available: http://www.pubmedcentral.nih.gov/articlerender.fcgi?artid=1745174&tool=pmcentrez&rendertype=abstract 9659348 10.1136/thx.53.3.159 PMC1745174

[16] Kalin M, Kanclerski K, Granström M, Möllby R. Diagnosis of pneumococcal pneumonia by enzyme-linked immunosorbent assay of antibodies to pneumococcal hemolysin (pneumolysin). J Clin Microbiol. 1987;25: 226–9. Available: http://www.pubmedcentral.nih.gov/articlerender.fcgi?artid=265872&tool=pmcentrez&rendertype=abstract 3818919 10.1128/jcm.25.2.226-229.1987 PMC265872

[17] Braun JS, Sublett JE, Freyer D, Mitchell TJ, Cleveland JL, Tuomanen EI, et al Pneumococcal pneumolysin and H 2 O 2 mediate brain cell apoptosis during meningitis. J Clin Invest. 2002;109: 19–27. 11781347 10.1172/JCI12035 PMC150815

[18] Paton JC, Rowan-Kelly B, Ferrante a. Activation of human complement by the pneumococcal toxin pneumolysin. Infect Immun. 1984;43: 1085–7. Available: http://www.pubmedcentral.nih.gov/articlerender.fcgi?artid=264298&tool=pmcentrez&rendertype=abstract 6698602 10.1128/iai.43.3.1085-1087.1984 PMC264298

[19] Boulnois GJ, Paton JC, Mitchell TJ, Andrew PW. Structure and function of pneumolysin, the multifunctional, thiol-activated toxin of Streptococcus pneumoniae. Mol Microbiol. 1991;5: 2611–2616. 1779752 10.1111/j.1365-2958.1991.tb01969.x

[20] Kadioglu A, Gingles NA, Grattan K, Kerr A, Mitchell TJ, Andrew PW, et al Host Cellular Immune Response to Pneumococcal Lung Infection in Mice Host Cellular Immune Response to Pneumococcal Lung Infection in Mice. Infect Immun. 2000;68: 492–501. 10639409 10.1128/iai.68.2.492-501.2000 PMC97168

[21] Mitchell TJ, Alexander JE, Morgan PJ, Andrew PW. Molecular analysis of virulence factors of Streptococcus pneumoniae. Soc Appl Bacteriol Symp Ser. 1997;26: 62S–71S. Available: http://www.ncbi.nlm.nih.gov/pubmed/9436318 9436318

[22] Camara M, Boulnois GJ, Andrew PW, Mitchell TJ. A neuraminidase from Streptococcus pneumoniae has the features of a surface protein. Infect Immun. 1994;62: 3688–3695. 8063384 10.1128/iai.62.9.3688-3695.1994 PMC303019

[23] Manco S, Hernon F, Yesilkaya H, Paton JC, Andrew PW, Kadioglu A. Pneumococcal neuraminidases A and B both have essential roles during infection of the respiratory tract and sepsis. Infect Immun. 2006;74: 4014–20. 10.1128/IAI.01237-05 16790774 PMC1489734

[24] Mochan E, Swigon D, Ermentrout GB, Lukens S, Clermont G. A mathematical model of intrahost pneumococcal pneumonia infection dynamics in murine strains. J Theor Biol. Elsevier; 2014;353: 44–54. 10.1016/j.jtbi.2014.02.021 PMC4058322 24594373

[25] Jounblat R, Kadioglu A, Mitchell TJ, Peter W, Andrew PW. Pneumococcal Behavior and Host Responses during Bronchopneumonia Are Affected Differently by the Cytolytic and Complement-Activating Activities of Pneumolysin. Infect Immun. 2003;71: 1813–1819. 12654795 10.1128/IAI.71.4.1813-1819.2003 PMC152068

[26] McCluskey J, Hinds J, Husain S, Witney A, Mitchell TJ. A two-component system that controls the expression of pneumococcal surface antigen A (PsaA) and regulates virulence and resistance to oxidative stress in Streptococcus pneumoniae. Mol Microbiol. 2004;51: 1661–1675. 15009893 10.1111/j.1365-2958.2003.03917.x

[27] Alexander JE, Berry a M, Paton JC, Rubins JB, Andrew PW, Mitchell TJ. Amino acid changes affecting the activity of pneumolysin alter the behaviour of pneumococci in pneumonia. Microb Pathog. 1998;24: 167–74. 10.1006/mpat.1997.0185 9514638

[28] Dalia AB, Standish AJ, Weiser JN. Three surface exoglycosidases from Streptococcus pneumoniae, NanA, BgaA, and StrH, promote resistance to opsonophagocytic killing by human neutrophils. Infect Immun. 2010;78: 2108–16. 10.1128/IAI.01125-09 20160017 PMC2863504

[29] Orihuela CJ, Gao G, Francis KP, Yu J, Tuomanen EI. Tissue-specific contributions of pneumococcal virulence factors to pathogenesis. J Infect Dis. 2004;190: 1661–9. 10.1086/424596 15478073

[30] Lanie J a, Ng W-L, Kazmierczak KM, Andrzejewski TM, Davidsen TM, Wayne KJ, et al Genome sequence of Avery’s virulent serotype 2 strain D39 of Streptococcus pneumoniae and comparison with that of unencapsulated laboratory strain R6. J Bacteriol. 2007;189: 38–51. 10.1128/JB.01148-06 17041037 PMC1797212

[31] Brooks-Walter A, Briles DE, Hollingshead SK. The pspC Gene of Streptococcus pneumoniae Encodes a Polymorphic Protein, PspC, Which Elicits Cross-Reactive Antibodies to PspA and Provides Immunity to Pneumococcal Bacteremia. Infect Immun. 1999;67: 6533–6542. 10569772 10.1128/iai.67.12.6533-6542.1999 PMC97064

[32] Read RC, Wilson R, Rutman A, Lund V, Todd HC, Brain APR, et al Interaction of Nontypable Haemophilus influenzae with Human Respiratory Mucosa In Vitro. J Infect Dis. 1991;163: 549–558. 1671682 10.1093/infdis/163.3.549

[33] Sethi S, Murphy TF. Bacterial Infection in Chronic Obstructive Pulmonary Disease in 2000 : a State-of-the-Art Review. Clin Microbiol Rev. 2001;14: 336–363. 11292642 10.1128/CMR.14.2.336-363.2001 PMC88978

[34] Mizgerd BJP, Meek BB, Kutkoski GJ, Bullard DC, Beaudet AU, Doerschuk CM. Selectins and Neutrophil Traffic: Margination and Streptococcus Pneumoniae-induced Emigration in Murine Lungs. J Exp Med. 1996;184: 639–645. 8760817 10.1084/jem.184.2.639 PMC2192708

[35] Spencer H. Interstitial pneumonia. Annu Rev Med. 1967;18: 423–42. 10.1146/annurev.me.18.020167.002231 5337533

[36] Metropolis N, Rosenbluth AW, Rosenbluth MN, Teller AH, Teller E. Equation of State Calculations by Fast Computing Machines. J Chem Phys. 1953;21: 1087 10.1063/1.1699114

[37] Hastings W. Monte Carlo sampling methods using Markov chains and their applications. Biometrika. 1970;57: 97–109.

[38] Earl DJ, Deem MW. Parallel tempering: theory, applications, and new perspectives. Phys Chem Chem Phys. 2005;7: 3910–6. Available: http://www.ncbi.nlm.nih.gov/pubmed/19810318 19810318 10.1039/b509983h

[39] Gelman A, Rubin D. Inference from iterative simulation using multiple sequences. Stat Sci. 1992;7: 457–511.

[40] Geweke J. Evaluating the Accuracy of Sampling-Based Approaches to the Calculation of Posterior Moments Bayesian Statistics 4. University Press; 1992.

